# Prognostic nutritional index for predicting the clinical outcomes of patients with gastric cancer who received immune checkpoint inhibitors

**DOI:** 10.3389/fnut.2022.1038118

**Published:** 2022-11-10

**Authors:** Hao Sun, Li Chen, Rong Huang, Hongming Pan, Yanjiao Zuo, Ruihu Zhao, Yingwei Xue, Hongjiang Song

**Affiliations:** ^1^Department of Gastrointestinal Surgery, Harbin Medical University Cancer Hospital, Harbin Medical University, Harbin, China; ^2^Department of Thyroid and Breast Surgery, Tongji Hospital, Tongji Medical College of Huazhong University of Science and Technology, Wuhan, China

**Keywords:** prognostic nutritional index, gastric cancer, immune checkpoint inhibitors, clinical outcomes, PD-1/PD-L1

## Abstract

**Objective:**

Although the application of immunotherapy in gastric cancer has achieved satisfactory clinical effects, many patients have no response. The aim of this retrospective study is to investigate the predictive ability of the prognostic nutrition index (PNI) to the prognosis of patients with gastric cancer who received immune checkpoint inhibitors (ICIs).

**Materials and methods:**

Participants were 146 gastric cancer patients with ICIs (PD-1/PD-L1 inhibitors) or chemotherapy. All patients were divided into a low PNI group and a high PNI group based on the cut-off evaluated by the receiver operating characteristic (ROC) curve. We contrasted the difference in progression-free survival (PFS) and overall survival (OS) in two groups while calculating the prognosis factors for PFS and OS by univariate and multivariate analyses. Moreover, the nomogram based on the results of the multivariate analysis was constructed to estimate the 1- and 3-year survival probabilities.

**Results:**

There were 41 (28.1%) cases in the low PNI group and 105 (71.9%) cases in the high PNI group. The median survival time for PFS in the low PNI group and high PNI group was 12.30 months vs. 33.07 months, and 18.57 months vs. not reached in the two groups for OS. Patients in low PNI group were associated with shorter PFS and OS in all patients [Hazard ratio (HR) = 1.913, *p* = 0.013 and HR = 2.332, *p* = 0.001]. Additionally, in subgroup analysis, low PNI group cases also had poorer PFS and OS, especially in patients with ICIs. In addition, the multivariate analysis found that carbohydrate antigen 724 (CA724) and TNM stage were independent prognostic factors for PFS. At the same time, indirect bilirubin (IDBIL), CA724, PNI, and TNM stage were independent prognostic factors for OS.

**Conclusion:**

Prognostic nutrition index was an accurate inflammatory and nutritional marker, which could predict the prognosis of patients with gastric cancer who received ICIs. PNI could be used as a biomarker for ICIs to identify patients with gastric cancer who might be sensitive to ICIs.

## Introduction

Because of the increase in population, although incidence rate and mortality have decreased, gastric cancer remains the fifth most diagnosed cancer, especially in East Asian countries such as China, Japan, and Korea. Gastric cancer is even the most common cancer in Korean and Japanese men (1). The treatment of patients with advanced gastric cancer is still a major challenge. Even after receiving radical resection and adjuvant therapy, patients’ 5-year survival rates remain low (2–5). Therefore, it is significantly important for patients with gastric cancer to find new treatments that can prolong survival time. Immune checkpoint inhibitors (ICIs) have been found to achieve favorable results in a variety of solid tumors, including triple-negative breast cancer, non-small cell lung cancer, and head and neck cancer (6–10). Therefore, people have focused on the application of ICIs in patients with gastric cancer. However, although the use of ICIs has brought benefits to patients with gastric cancer, some cases were not sensitive to ICIs, even if they were patients with PD-1/PD-L1 positive and microsatellite instability-high (MSI-H) (11, 12). At the same time, some studies have also shown that gastric cancer patients with PD-1/PD-L1 negative and microsatellite stable (MSS) might still benefit from ICIs (13, 14). Therefore, exploring a simple and accurate biomarker to predict patients with gastric cancer who can benefit from ICIs is necessary.

Many studies have shown that nutritional status was related to cancer prognosis (15–20). Due to the especially anatomical characteristics, nutritional status has a more obvious impact on patients with gastric cancer (21–24). Nutritional markers such as albumin, prealbumin, and body mass index have been found to be independent prognostic factors for gastric cancer (25). A systemic inflammatory state can lead to cancer metastasis and progression. Previous studies have shown that peripheral inflammatory markers such as lymphocytes, neutrophils, and C-reactive proteins were also related to the prognosis of patients with gastric cancer (26). In a word, gastric cancer patients with malnutrition and system inflammation status tended to have higher metastasis rates and shorter survival times. Numerous previous studies have shown that composite indexes based on serum markers such as the Controlling Nutritional Status (CONUT) score, Glasgow Prognostic Score (GPS), and neutrophil-lymphocyte ratio (NLR) could both predict the prognosis of patients with gastric cancer (27–29). In addition, their ability to predict the prognosis of patients with gastric cancer who received ICIs has also been further confirmed.

The prognostic nutrition index (PNI) is calculated by serum albumin and lymphocyte. Current studies have shown that PNI has high accuracy in predicting the prognosis of various cancers after treatment, especially gastric cancer (30, 31). The immune system function is an important cause of ICI’s works, and serum albumin and lymphocyte in the bloodstream play an important role in reflecting immune system function. However, there has been still no evidence to demonstrate the usefulness of PNI as a prognosis assessment tool for patients with gastric cancer who received ICIs.

In this study, we enrolled 146 patients with gastric cancer at our institution who received ICIs (PD-1/PD-L1 inhibitors) or chemotherapy, and primarily analyzed the predictive ability of PNI for the prognosis of patients with ICIs. At the same time, we also performed subgroup analysis and constructed a nomogram to further test and verify the effectiveness of PNI in predicting the prognosis of patients with gastric cancer.

## Materials and methods

### Patients

There were 146 patients with gastric cancer treated with chemotherapy or ICIs at our institution from August 2016 to December 2020 enrolled in this study. All patients and their clinical information were analyzed based on the Helsinki Declaration as well as its amendments and included according to the following criteria: (1) all patients underwent invasive gastroscope examination and pathological diagnosis; (2) all patients had no chronic disease or other types of cancer; and (3) all patients were received ICIs or chemotherapy. Patients with incomplete clinical information and without a regular review after treatment or abandoned treatment were exclusion criteria. An electronic medical record system was used to collect clinical and pathological information. Informed consent was waived by the Ethics Committee of Harbin Medical University Cancer Hospital owing to the retrospective nature of this study.

### Data collection

Routine telephone follow-up was used for all enrolled patients. Clinical and pathological information was acquired by an electronic medical record system. Progression-free survival (PFS) and overall survival (OS) were obtained by follow-up. PFS was comprehended as the period from the first day of surgery, ICIs, or chemotherapy date to the date of disease progression. The evidence of progression was obtained by chest and abdomen X-ray or computed tomography. PFS was also defined at the date of death or last follow-up with no evidence of progression in patients. OS was described as the period from the first day of ICIs, surgery, or chemotherapy date to the date of death or the last follow-up. PNI was calculated as follows: PNI = albumin (g/L) + 5 × lymphocyte (10^9^/L). The cut-off point was obtained by receiver operating characteristic (ROC), and patients were divided into low-value groups (PNI < 44.63) and high-value groups (PNI ≥ 44.63) according to the cut-off point.

### Statistical analysis

All statistical analyses were completed through the SPSS software 25.0 (Chicago, IL, USA) and R 4.1.3 (Vienna, Austria). Two-sided *P*-values < 0.05 were considered as statistical differences. The Chi-square test or Fisher’s exact test was used to compare the discrepancies between the two groups, the Kaplan–Meier survival curve was used to compute the survival rate, and the Log-rank test was used to compare the difference in survival time. Relative risks were assessed by the hazard ratio (HR) and 95% confidence interval (CI). The independent prognostic factors were analyzed by the constructed Cox proportional hazards regression model. Finally, the nomogram based on independent prognostic factors was established to predict the survival probability of PFS and OS.

## Results

### Patient characteristics

According to the cut-off value of PNI, 41 (28.1%) patients in this study entered the low PNI group and 105 (71.9%) patients were in the high PNI group. The median age of patients was 59 years, and there were 44 women (30.1%) and 102 men (69.9%) in all two groups’ cases. In this study, 86 (58.9%) patients received surgery plus adjuvant therapy and 60 (41.1%) patients received only adjuvant therapy. Chi-square test shown that PNI was related to age (*p* = 0.011), electrocardiogram (*p* = 0.006), pathology (*p* = 0.008), and PD-L1 (*p* = 0.046). The detailed clinical characteristics of all 146 cases grouped by PNI are shown in Table 1.

**TABLE 1:** The clinical information of all patients.

	Level	Low PNI	High PNI	*p*
		
N		41	105	
Sex	Male	31	71	0.344
	Female	10	34	
Age	< 59	26	42	0.011
	≥ 59	15	63	
BMI	< 21.55	23	50	0.357
	≥ 21.55	18	55	
ABO blood type	A + B	24	62	0.955
	O + AB	14	43	
SLNM	No	37	91	0.555
	Yes	4	14	
ECG	Normal	23	81	0.012
	Abnormal	18	24	
Surgery	Yes	22	64	0.421
	No	19	41	
Primary tumor site	Upper 1/3	7	14	0.825
	Middle 1/3	14	31	
	Low 1/3	17	52	
	Whole	3	8	
Borrmann type	Borrmann I + II	3	6	0.623
	Borrmann III + IV	19	58	
	Unknown	19	41	
Tumor size (mm)	< 50	12	29	0.844
	≥ 50	10	22	
	Unknown	19	54	
Differentiation	Poor	25	70	0.065
	Moderately + Well	10	31	
	Unknown	6	4	
Pathology	Adenocarcinoma	25	72	0.007
	Others^#^	8	29	
	Unknown	8	4	
TNM stage	I + II	5	19	0.387
	III + IV	36	86	
Lauren type	Intestinal	7	26	0.453
	Diffuse	4	17	
	Mixed	7	16	
	Unknown	23	46	
PD-1	Negative	17	48	0.211
	Positive	2	14	
	Unknown	22	43	
PD-L1	Negative	14	28	0.046
	Positive	5	34	
	Unknown	22	43	
Treatment	ICIs	27	62	0.449
	Chemotherapy	14	43	

^#^BMI, body mass index; ECG, electrocardiogram; SLNM, supraclavicular lymph node; Others of Pathology, include mucinous carcinoma, signet ring cell carcinoma, mixed carcinoma, unknown.

### Blood parameters

In this study, we analyzed the blood examination results of patients before treatment and studied their relationship to PNI by Chi-square test or Fisher’s exact test. We grouped patients according to the medians of total protein (TP), albumin (ALB), globulin (GLOB), prealbumin (PALB), total bilirubin (TBIL), direct bilirubin (DBIL), indirect bilirubin (IDBIL), lymphocyte (L), hemoglobin (Hb), lymphocyte carcinoembryonic antigen (CEA), carbohydrate antigen 199 (CA199), carbohydrate antigen 724 (CA724), and carbohydrate antigen 125II (CA125II). Their medians and detailed blood parameters are shown in Table 2. We found that PNI was related to TP (*p* < 0.001), PALB (*p* = 0.001), and Hb (*p* < 0.001). At the same time, patients in the low PNI group were found to have lower ALB (*p* < 0.001) and L (*p* < 0.001) than the high PNI group by Fisher’s exact test.

**TABLE 2:** The blood parameters of all patients.

	level	Low PNI	High PNI	*p*
		
*n*		41	105	
TP (g/L)	< 68.70	31	41	< 0.001
	≥ 68.70	10	64	
ALB (g/L)	< 38.95	38	35	< 0.001
	≥ 38.95	3	70	
GLOB (g/L)	< 29.10	25	48	0.097
	≥ 29.10	16	57	
PALB (mg/L)	< 200	29	43	0.001
	≥ 200	12	62	
TBIL (μmol/L)	< 12.10	21	51	0.774
	≥ 12.10	20	54	
DBIL (μmol/L)	< 2.72	23	50	0.357
	≥ 2.72	18	55	
IDBIL (μmol/L)	< 8.88	21	52	0.854
	≥ 8.88	20	53	
L (10^9^/L)	< 1.70	38	35	< 0.001
	≥ 1.70	3	70	
Hb (g/L)	< 122.50	30	43	< 0.001
	≥ 122.50	11	62	
CEA (ng/ml)	< 2.54	18	55	0.357
	≥ 2.54	23	50	
AFP (ng/ml)	< 3.02	21	52	0.854
	≥ 3.02	20	53	
CA199 (U/ml)	< 14.40	17	55	0.236
	≥ 14.40	24	50	
CA724 (U/ml)	< 2.56	18	55	0.357
	≥ 2.56	23	50	
CA125II (U/ml)	< 27.59	18	55	0.357
	≥ 27.59	23	50	

### Univariate and multivariate Cox hazard analyses for progression-free survival and overall survival

According to univariate analysis, the prognosis factors of patients in this study for PFS and OS were both IDBIL (*p* = 0.046 vs. *p* = 0.032), PALB (*p* = 0.013 vs. *p* = 0.006), PNI (*p* = 0.013 vs. *p* = 0.001), CEA (*p* = 0.037 vs. *p* = 0.004), CA199 (*p* = 0.032 vs. *p* = 0.009), CA724 (*p* = 0.003 vs. *p* = 0.001), radical resection (*p* = 0.001 vs. *p* < 0.001), surgery (*p* = 0.005 vs. *p* = 0.006), Borrmann type (*p* = 0.040 vs. *p* = 0.040), TNM stage (*p* = 0.004 vs. *p* = 0.001), Lauren type (*p* = 0.003 vs. *p* = 0.002), and treatment (*p* = 0.001 vs. *p* < 0.001). In addition, TBIL (*p* = 0.042) was also found to be the prognosis factor for PFS by univariate analysis. The multivariate analysis indicated that CA724 (*p* = 0.028 vs. *p* = 0.019) and TNM stage (*p* = 0.019 vs. *p* = 0.010) were both independent prognostic factors for PFS and OS. In addition, PNI (*p* = 0.030) and IDBIL (*p* = 0.032) were also the independent prognostic factors for OS. The detailed information is shown in Table 3.

**TABLE 3:** Univariate and multivariate analyses for progression-free survival (PFS) and overall survival (OS).

	PFS				OS			
	Univariate analysis		Multivariate analysis		Univariate analysis		Multivariate analysis	
	
Parameters	Hazard ratio (95% CI)	*P*-value	Hazard ratio (95% CI)	*P*-value	Hazard ratio (95% CI)	*P*-value	Hazard ratio (95% CI)	*P*-value
Sex (Male vs. Female)	1.099 (0.645–1.872)	0.729			1.084 (0.636–1.846)	0.768		
Age (< 59 vs. ≥ 59)	0.914 (0.555–1.505)	0.724			0.876 (0.532–1.442)	0.603		
TP (< 68.70 g/L vs. ≥ 68.70 g/L)	0.842 (0.511–1.386)	0.498			0.789 (0.478–1.299)	0.351		
GLOB (< 29.10 g/L vs. ≥ 29.10 g/L)	1.140 (0.691–1.881)	0.608			1.245 (0.755–2.054)	0.391		
BMI (< 21.55 vs. ≥ 21.55)	0.865 (0.526–1.424)	0.569			0.871 (0.529–1.434)	0.588		
TBIL (< 12.10 μmol/L vs. ≥ 12.10 μmol/L)	1.706 (1.020–2.853)	0.042	1.362 (0.493–3.764)	0.551	1.564 (0.938–2.610	0.087		
DBIL (< 2.72 μmol/L vs. ≥ 2.72 μmol/L)	1.131 (0.681–1.875)	0.635			1.084 (0.625–1.803)	0.756		
IDBIL (< 8.88 μmol/L vs. ≥ 8.88 μmol/L)	1.680 (1.008–2.800)	0.046	1.204 (0.433–3.346)	0.721	1.742 (1.047–2.899)	0.032	1.899 (1.055–3.417)	0.032
PALB (< 200 mg/L vs. ≥ 200 mg/L)	0.523 (0.313–0.872)	0.013	0.601 (0.347–1.042)	0.070	0.485 (0.291–0.811)	0.006	0.640 (0.370–1.106)	0.110
ALB (< 38.95 g/L vs. ≥ 38.95 g/L)	0.803 (0.487–1.324)	0.390			0.776 (0.471–1.280)	0.320		
PNI (< 44.63 vs. ≥ 44.63)	1.913 (1.145–3.196)	0.013	1.359 (0.762–2.424)	0.298	2.332 (1.393–3.905)	0.001	1.860 (1.061–3.260)	0.030
ABO blood type (A + B vs. AB + O)	1.449 (0.880–2.388)	0.145			1.404 (0.853–2.311)	0.182		
Hb (< 122.5 g/L vs. ≥ 122.5 g/L)	1.105 (0.672–1.819)	0.694			1.051 (0.639–1.730)	0.844		
CEA (< 2.54 U/ml vs. ≥ 2.54 U/ml)	1.725 (1.034–2.877)	0.037	1.117 (0.615–2.030)	0.716	2.154 (1.282–3.618)	0.004	1.253 (0.690–2.278)	0.459
AFP (< 3.02 U/ml vs. ≥ 3.02 U/m)	0.787 (0.477–1.298)	0.348			0.832 (0.505–1.373)	0.472		
CA199 (< 14.40 U/ml vs. ≥ 14.40 U/ml)	1.752 (1.050–2.923)	0.032	1.194 (0.704–2.026)	0.510	1.973 (1.181–3.298)	0.009	1.168 (0.683–1.995)	0.571
CA724 (< 2.56 U/ml vs. ≥ 2.56 U/ml)	2.245 (1.326–3.802)	0.003	1.859 (1.067–3.237)	0.028	2.370 (1.398–4.018)	0.001	1.958 (1.115–3.437)	0.019
CA125II (< 27.59 U/ml vs. ≥ 27.59 U/ml)	1.237 (0.751–2.039)	0.404			1.168 (0.709–1.924)	0.542		
Radical resection (R0 vs. non R0)	2.501 (1.478–4.231)	0.001	1.661 (0.658–4.191)	0.282	2.800 (1.631–4.807)	< 0.001	2.016 (0.839–4.848)	0.117
Surgery (yes vs. no)	2.069 (1.242–3.446)	0.005	1.079 (0.467–2.494)	0.859	2.093 (1.242–3.526)	0.006	1.411 (0.598–3.325)	0.432
Borrmann type (I + II vs. III + IV + Unknown)	1.625 (1.023–2.583)	0.040	1.649 (0.592–4.594)	0.338	1.636 (1.023–2.616)	0.040	1.765 (0.639–4.873)	0.273
Tumor size (< 50 mm vs. ≥ 50 mm + Unknown)	1.131 (0.847–1.511)	0.405			1.140 (0.853–1.523)	0.377		
TNM stage (I + II vs. III + IV)	5.531 (1.731–17.674)	0.004	4.209 (1.271–13.935)	0.019	6.605 (2.060–21.181)	0.001	4.796 (1.444–15.930)	0.010
Lauren type (intestinal vs. others^#^)	1.415 (1.125–1.781)	0.003	1.098 (0.765–1.576)	0.610	1.426 (1.133–1.796)	0.002	1.081 (0.755–1.546)	0.671
Treatment (ICIs vs. chemotherapy)	2.812 (1.558–5.074)	0.001	1.404 (0.716–2.753)	0.323	3.078 (1.696–5.586)	< 0.001	1.337 (0.686–2.608)	0.394

^#^Others of Lauren type were diffuse, mixed, and unknown.

### Survival for prognostic nutrition index

**FIGURE 1:**
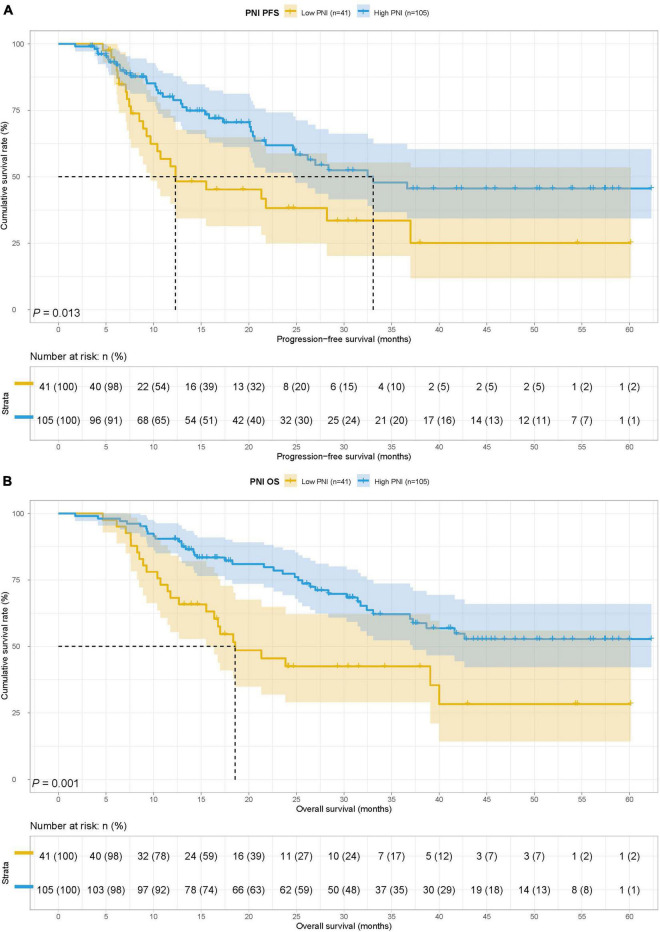
Prognostic nutrition index (PNI) related survival curve of **(A)** progression-free survival (PFS) and **(B)** overall survival (OS) in all patients.

The low PNI group’s median survival time (MST) for PFS and OS was 12.30 months and 18.57 months, while the MST for PFS and OS in the high PNI group was 33.07 months and not reached. Low PNI group’s patients had shorter PFS and OS than patients in the high PNI group (HR = 1.913, 95% CI: 1.145–3.196, *p* = 0.013 and HR = 2.332, 95% CI: 1.393–3.905, *p* = 0.001) (Figures 1A,B).

### Survival for treatment

**FIGURE 2:**
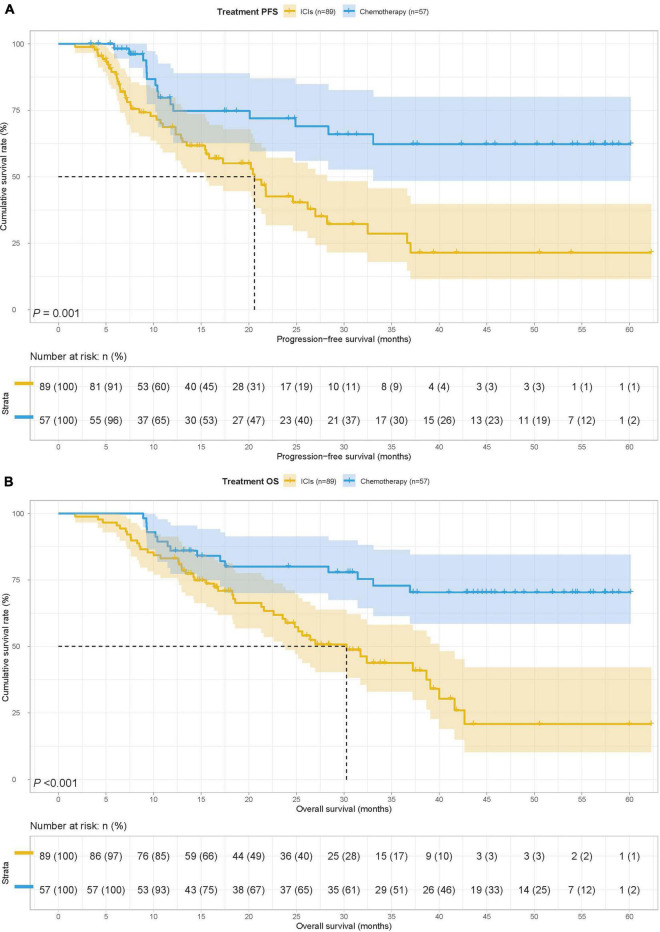
Treatment related survival curve of **(A)** PFS and **(B)** OS in all patients.

To study the predictive ability of PNI for the prognosis of gastric cancer patients with ICIs, we divided the 146 cases into the ICIs group (89 patients) and the chemotherapy group (57 patients). Treatment was related to surgery (*p* < 0.001), tumor size (*p* = 0.046), TNM stage (*p* = 0.002), and Lauren type (*p* < 0.001). At the same time, Fisher’s exact test found that treatment was also related to Borrmann type (*p* < 0.001), PD-1 (*p* < 0.001), and PD-L1 (*p* < 0.001) (Table 4). The MST for PFS and OS in the ICIs group was 20.60 months and 30.27 months, and the MST for PFS and OS in the chemotherapy group was both not reached. Patients received chemotherapy had longer PFS (HR = 0.356, 95% CI: 0.197–0.642, *P* = 0.001) and OS (HR = 0.325, 95% CI: 0.179–0.590, *p* < 0.001) than those received ICIs (Figures 2A,B).

**TABLE 4:** The clinical information for the treatment.

	Level	ICIs	Chemotherapy	*p*
		
*n*		89	57	
Age	< 59	42	26	0.852
	≥ 59	47	31	
SLNM	No	75	53	0.118
	Yes	14	4	
Surgery	Yes	38	48	< 0.001
	No	51	9	
Primary tumor site	Upper 1/3	14	7	0.902
	Middle 1/3	28	17	
	Low 1/3	41	28	
	Whole	6	5	
Borrmann type	Borrmann I + II	6	3	< 0.001
	Borrmann III + IV	32	45	
	Unknown	51	9	
Tumor size	< 50 mm	19	22	0.046
	≥ 50 mm	19	13	
	Unknown	51	22	
TNM stage	I + II	8	16	0.002
	III + IV	81	41	
Lauren type	Intestinal	14	19	< 0.001
	Diffuse	8	13	
	Mixed	8	15	
	Unknown	59	10	
PD-1	Negative	18	47	< 0.001
	Positive	6	10	
	Unknown	65	0	
PD-L1	Negative	8	34	< 0.001
	Positive	16	23	
	Unknown	65	0	

**FIGURE 3:**
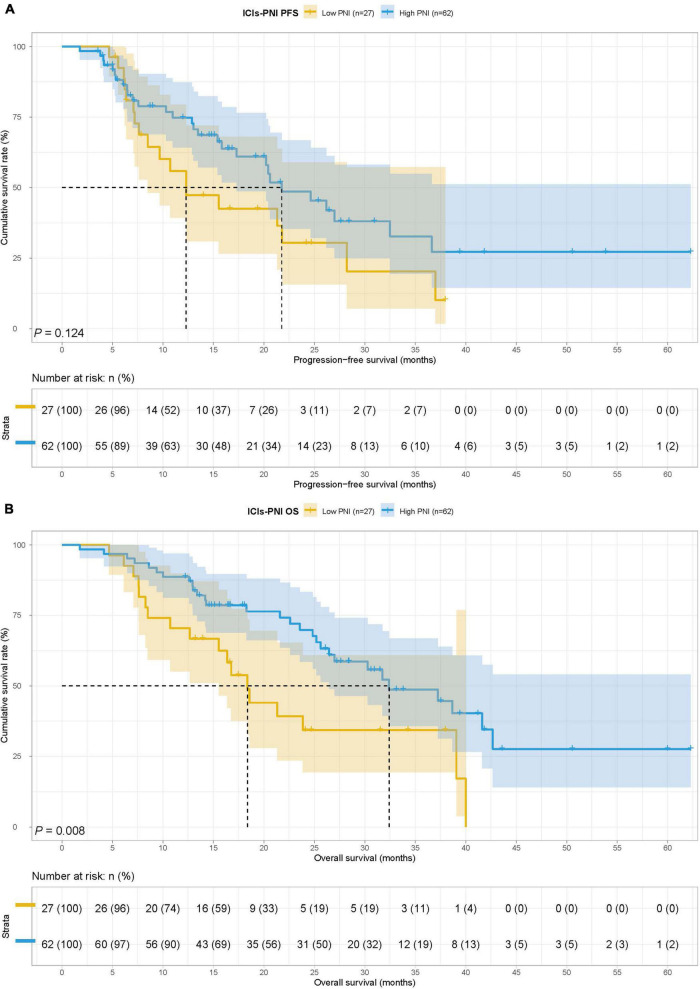
Prognostic nutrition index related survival curve of **(A)** PFS and **(B)** OS in the ICIs group.

In the ICIs group, there were 27 cases in the low PNI group and 62 cases in the high PNI group, The MST for PFS in the low PNI group and high PNI group was 12.30 months vs. 21.77 months, while the MST for OS in the two groups was 18.37 vs. 32.40 months. Low PNI group had poorer PFS (HR = 1.583, 95% CI: 0.877–2.857, *p* = 0.124) and OS (HR = 2.210, 95% CI: 1.209–4.040, *p* = 0.008) than high PNI group in patients with ICIs (Figures 3A,B).

**FIGURE 4:**
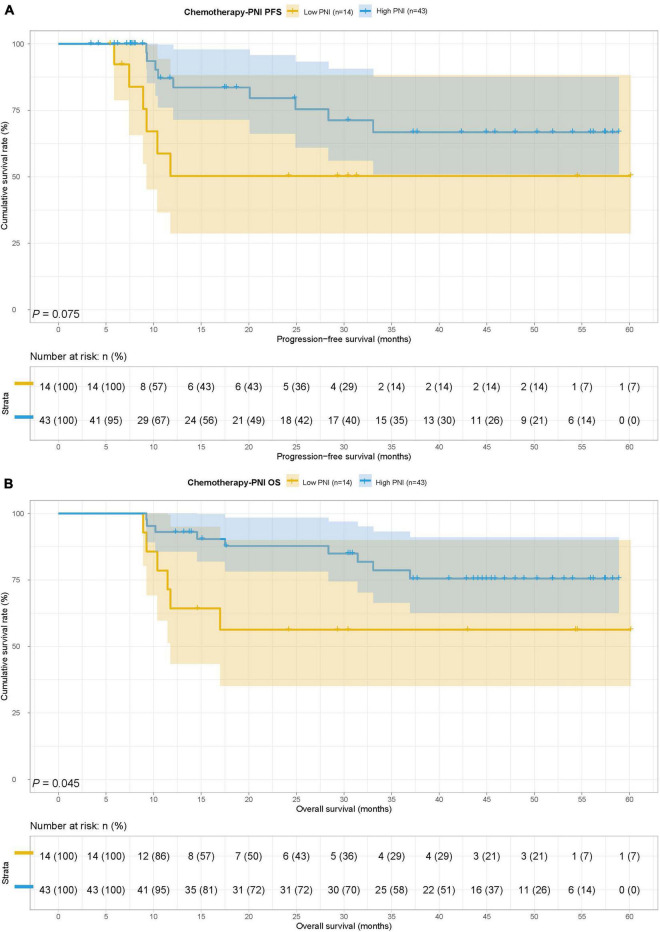
Prognostic nutrition index related survival curve of **(A)** PFS and **(B)** OS in the chemotherapy group.

In the chemotherapy group, there were 14 patients in the low PNI group and 43 patients in the high PNI group. The MST for PFS and OS in the low PNI group and high PNI group was both not reached. Low PNI group was also associated with shorter PFS and OS than high PNI group (HR = 2.489, 95% CI: 0.882–7.028, *p* = 0.075 and HR = 2.778, 95% CI: 0.981–7.869, *p* = 0.045) (Figures 4A,B).

### Survival for prognostic nutrition index in prealbumin

**FIGURE 5:**
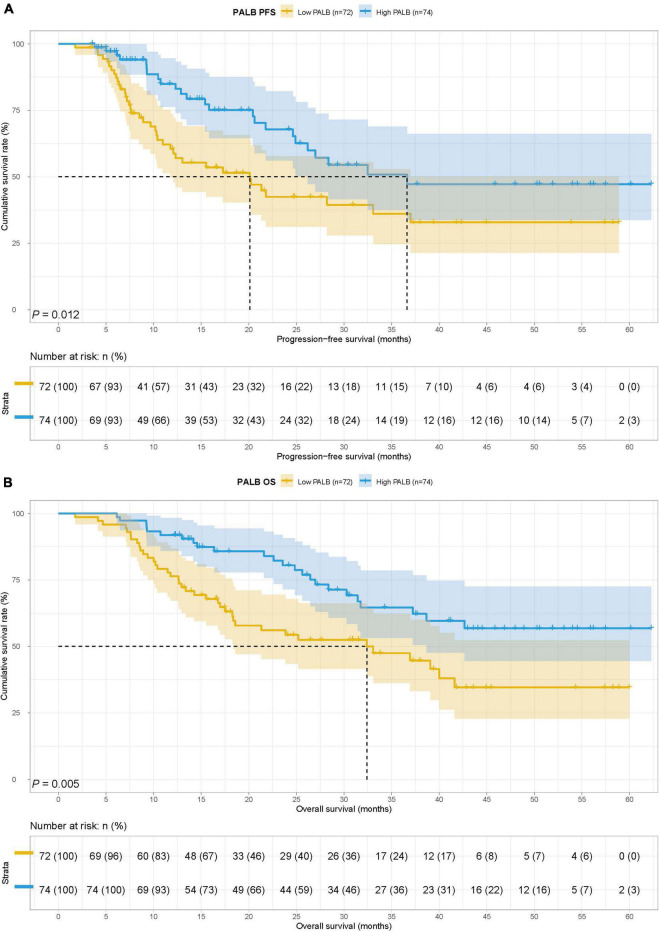
Prealbumin (PALB) related survival curve of **(A)** PFS and **(B)** OS in all patients.

In this study, we found that PALB was both PFS and OS’s independent prognostic factor by multivariate analysis. To further checked the prognostic prediction ability of PNI, we extra analyzed the application of PNI in gastric cancer patients with different PALB states. According to the median PALB value, patients in this study were enrolled in the low PALB group (PALB < 200) and high PALB group (PALB ≥ 200). The MST for PFS in the low PALB group and high PALB group was 20.10 months vs. 36.63 months, respectively, while the MST for OS in the two groups was 32.40 months vs. not reached. Patients with the low PALB group had shorter PFS and OS than high PALB group in this study (HR = 0.523, 95% CI: 0.313–0.872, *p* = 0.012 and HR = 0.485, 95% CI: 0.291–0.811, *p* = 0.005) (Figures 5A,B).

**FIGURE 6:**
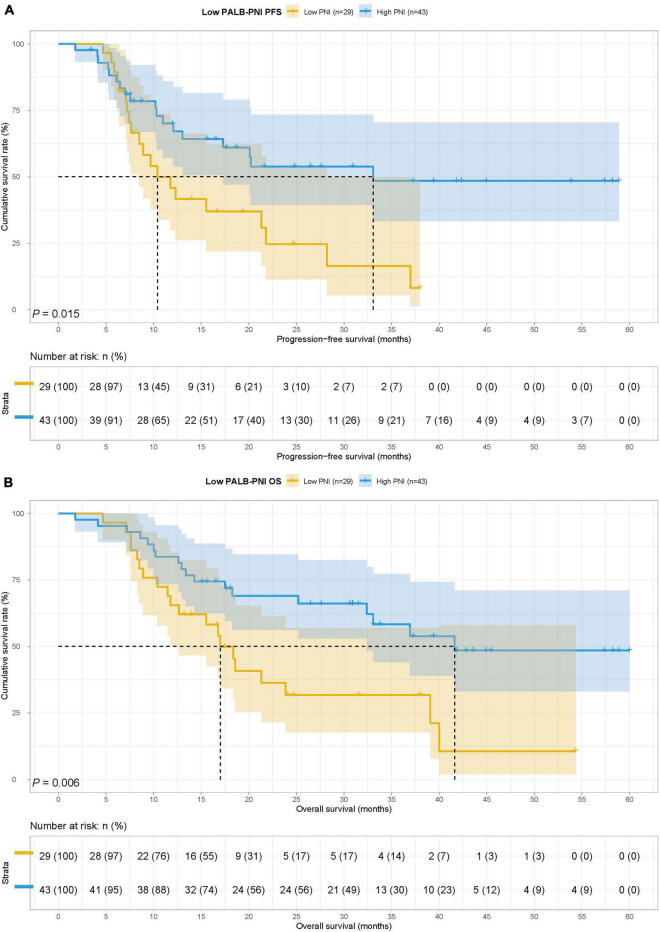
Prognostic nutrition index related survival curve of **(A)** PFS and **(B)** OS in the low PALB group.

In the low PALB group, there were 29 cases in the low PNI group and 43 cases in the high PNI group. The MST for PFS in the low PNI group and high PNI group was 10.40 months vs. 33.07 months, respectively, while the MST for OS in the two groups was 17.00 months vs. 41.63 months. Low PNI group’s patients had poorer PFS and OS than patients in the high PNI group in this study (HR = 2.191, 95% CI: 1.150–4.175, *p* = 0.015 and HR = 2.429, 95% CI: 1.268–4.653, *p* = 0.006) (Figures 6A,B).

**FIGURE 7:**
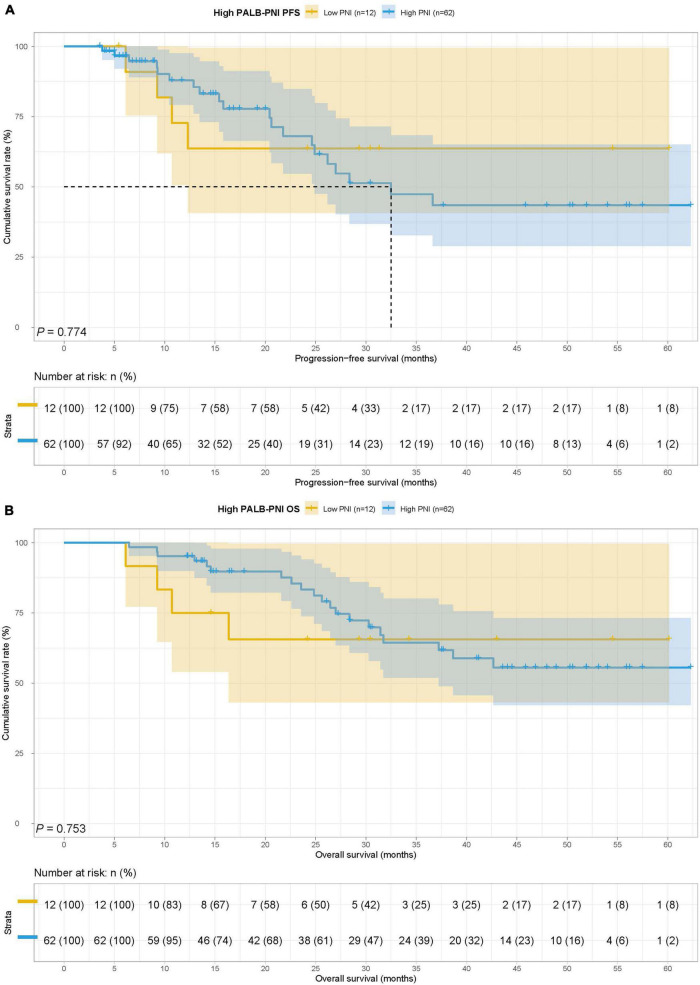
Prognostic nutrition index related survival curve of **(A)** PFS and **(B)** OS in the high PALB group.

In the high PALB group, there were 12 cases in the low PNI group and 62 cases in the high PNI group. The MST for PFS in the low PNI group and high PNI group was not reached vs. 28.37 months, while the MST for OS in the two groups was both not reached. There was no significant difference for PFS and OS in two groups (HR = 1.170, 95% CI: 0.399–3.433, *p* = 0.774 and HR = 0.841, 95% CI: 0.287–2.463, *p* = 0.753) (Figures 7A,B).

### Construction of nomograms to predict progression-free survival and overall survival

**FIGURE 8:**
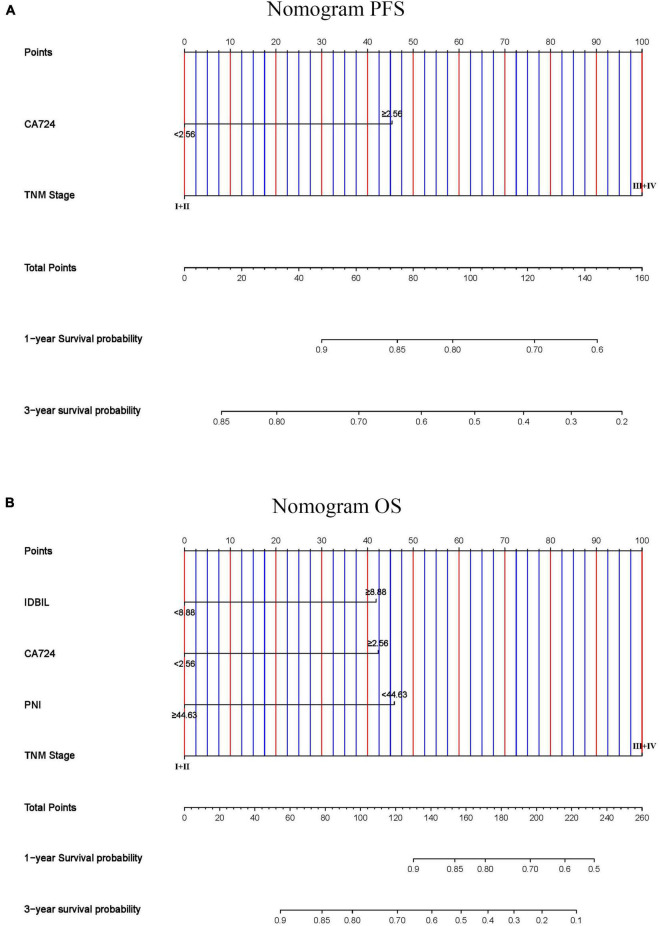
Nomogram for predicting 1- and 3-year survival probabilities of **(A)** PFS and **(B)** OS.

In this study, we found that CA724 and TNM stages were the independent prognostic factors for PFS and PNI, IDBIL, CA724, and TNM stages were the independent prognostic factors for OS by the constructed Cox proportional hazards regression model. According to the results of multivariate analysis, the nomograms to predict the 1- and 3-year survival probabilities for PFS and OS were established (Figures 8A,B). The C-index and 95% CI for predicting the survival probability of PFS and OS were 0.649 (0.588–0.710) and 0.737 (0.674–0.799).

## Discussion

With the wide application of ICIs in gastric cancer, people began to seek accurate biomarkers to predict the sensitivity of patients with gastric cancer to ICIs. However, current biomarkers include PD-1/PD-L1 expression levels, microsatellite instability (MSI), tumor mutation burden (TMB), and Epstein–Barr virus (EBV) infection status were still some limitations (11, 12, 32). The function of the immune system, which is affected by nutrition and inflammation, is one of the important factors for ICIs (33, 34). Therefore, we attempted to find a biomarker based on nutritional and inflammatory status to predict the clinical outcomes of patients with gastric cancer who received ICIs.

The PNI is a biomarker to evaluate the nutritional and inflammatory status of patients. In 1984, Onodera et al. first created it to evaluate the nutritional status of surgical patients, predict the surgical risk, and judge the prognosis (35). In subsequent studies, the predictive role of PNI in various tumors was gradually discovered. Okadome et al. analyzed 337 patients with esophageal cancer and found that patients with low PNI values were significantly correlated with clinical outcomes. Low PNI group patients had shorter OS in their study (36). The studies of Bozkaya et al. and Li et al. on 280 patients with prostate cancer and 333 patients with metastatic non-small cell lung cancer obtained similar results. The low PNI group had poorer clinical outcomes (37, 38). At the same time, people found that PNI was more accurate in predicting the prognosis of patients with gastric cancer. In a retrospective study of 245 gastric cancer patients with total gastrectomy, Xishan et al. found that patients with low PNI status were closely correlated with poor prognosis by univariate analysis. Multivariate analysis also found that PNI was an independent prognostic factor for patients with gastric cancer (39). Park et al. studied the correlation between PNI and BMI change before/after surgery and prognosis in patients with gastric cancer. They collected 1,868 patients with gastric cancer treated with gastrectomy and found that preoperative low BMI and PNI status as well as decreased BMI and PNI before/after surgery were all associated with poor prognosis. At the same time, BMI and PNI were both gastric cancer patients’ independent prognostic factors (30). Hirahara et al. retrospectively recruited 368 gastric cancer patients with normal serum CEA levels and analyzed the predictive ability of PNI for these subjects. The results showed that both univariate and multivariate analyses found that PNI was significantly correlated with the prognosis of gastric cancer patients with normal serum CEA levels, and patients with low PNI values had poorer cancer-specific survival (CSS) (40). To sum up, PNI has a strong predictive ability for prognosis in various cancers, especially gastric cancer.

This study mainly analyzed the use of PNI in gastric cancer patients with ICIs or chemotherapy. We also further explored the predictive ability of PNI on the prognosis of patients with gastric cancer in different PALB states. The results showed that patients in the low PNI group had shorter PFS and OS in all subjects. Similar results were also obtained in the ICIs group and chemotherapy group in subgroup analysis, especially the ICIs group. In the multivariate analysis, we found that ALB, PALB, PNI, CA724, and TNM stage were significant independent prognostic factors for both PFS and OS. Similarly, further analysis revealed that low PNI value patients had shorter survival time in the low PALB group. However, restricted by the number of patients, we did not get meaningful results in the analysis of the high PALB group.

In this study, we analyzed the effects of ICIs and chemotherapy on the clinical outcomes of patients with gastric cancer. The results showed that patients who received ICIs had poorer PFS and OS. This is because ICIs are still not routinely administered to patients with gastric cancer at our institution. In most cases, ICIs are only considered for patients with progressive gastric cancer whose conventional therapy has failed. The results of correlation analysis also reflect this phenomenon, and the use of ICIs was significantly related to non-surgery and high TNM stages, which were both significant independent prognostic factors for patients with gastric cancer according to previous studies.

The mechanism that caused this observation remains unclear. PNI contains albumin and lymphocyte, which reflect the nutrition and immune status of the body to a certain extent. On the one hand, low serum albumin represents malnutrition. On the other hand, previous studies have shown that albumin was also a biomarker for systemic inflammation (41). Some inflammatory factors can inhibit the synthesis of albumin, and oxidative stress can lead to the denaturation of albumin, all these factors lead to the rapid decrease of serum albumin levels in patients with the inflammation state (42, 43). The state of malnutrition and systemic inflammation were important factors leading to tumor progression (44–47). The lymphocyte is part of the immune system and has strong anti-tumor activity (48). Studies have shown that low serum lymphocyte is significantly associated with poor prognosis in patients with gastric cancer (49–51). ICIs against tumors by relieving the inhibition of tumor cells on the immune system, so nutrition and immune status are important factors for its working (52). Therefore, PNI combined with serum albumin and lymphocyte can effectively predict the efficacy of ICIs.

Finally, our study inevitably had some limitations. First, this was a single-center retrospective study, a prospective randomized controlled trial was an important method to overcome potential information bias. Second, the types of ICIs were not strictly distinguished; patients used different ICIs in this study. Third, the cut-off value of PNI was often calculated by the ROC curve, and there was still no recognized standard. Forth, as a systemic inflammation and nutrition evaluation index, PNI only included albumin and lymphocyte, which could be combined with more markers such as the Geriatric Nutritional Risk Index (GNRI) which reflect the changes in body weight. The result of this study also needs to be further verified by a study with larger sample sizes and better design.

## Conclusion

The PNI was an accurate prognostic tool for patients with gastric cancer. In patients with gastric cancer who received ICIs, it displayed a better ability to predict the prognosis. Patients with low PNI had poorer clinical outcomes. Therefore, PNI can be used as a biomarker for ICIs to identify patients with gastric cancer who are sensitive to ICIs.

## Data availability statement

The raw data supporting the conclusions of this article will be made available by the authors, without undue reservation.

## Ethics statement

The studies involving human participants were reviewed and approved by the Ethics Committee of Harbin Medical University Cancer Hospital. Written informed consent for participation was not required for this study in accordance with the national legislation and the institutional requirements.

## Author contributions

HSu, RH, and LC: writing—original draft, review, and editing. RZ and HP: data curation and investigation. YZ: methodology and supervision. YX and HSo: resources, funding acquisition, and project administration. All authors contributed to the article and approved the submitted version.
